# Dynamic Changes in Flavor-Related Compounds and Bacterial Community Structure During Sichuan Jiangrou Ripening

**DOI:** 10.3390/foods15183258

**Published:** 2026-09-15

**Authors:** Xiangling You, Tianyang Wang, Haibin Yuan, Wanting Tang, Huachang Wu, Xiangbo Xu

**Affiliations:** 1 Cuisine Science Key Laboratory of Sichuan Province, Sichuan Tourism University, Chengdu 610100, China; yxl19921225@163.com (X.Y.); 15883250273@163.com (H.Y.); twt15390399773@sina.com (W.T.); 2 College of Life Sciences and Agriculture & Forestry, Southwest University of Science and Technology, Mianyang 621000, China; wangtianyang@mails.swust.edu.cn; 3 Faculty of Food and Biological Engineering, Chengdu University, Chengdu 610106, China

**Keywords:** Sichuan Jiangrou, ripening process, flavoromics, bacterial community, machine learning

## Abstract

Ripening time is a major determinant of quality and flavor formation in traditional Sichuan fermented Jiangrou (JR). However, the changes in physicochemical properties, flavor compounds, and bacterial communities during JR ripening remain insufficiently understood. Therefore, this study aimed to characterize the dynamic changes in physicochemical properties, flavor-related compounds, and bacterial communities during JR ripening and to identify potential markers associated with different ripening stages. JR samples collected at eight ripening stages (0–35 days) were characterized using physicochemical measurements, electronic tongue analysis, free amino acid profiling, GC-MS, and 16S rRNA amplicon sequencing. The data were further analyzed using Spearman correlation analysis and interpretable machine learning. The results indicated that moisture content decreased from 74.60 to 45.87 g/100 g, whereas salt content increased to 3.67 g/100 g. Lipid oxidation progressively intensified, while superoxide dismutase activity and total free amino acids reached their highest levels around day 25. Total free amino acids increased from 31.85 to 313.98 mg/100 g before declining during late ripening. These temporal patterns suggest that days 20–25 represent a transition period under the conditions tested. Electronic tongue and volatile-profile analyses revealed pronounced stage-dependent changes in taste and aroma. Aldehydes accumulated prominently at day 25, whereas esters became predominant during late ripening. An XGBoost model built with aroma-active compounds correctly classified all samples in the current dataset. SHAP analysis identified heptanal, octanal, ethanol, methyl pentanoate, and 2-methylbutanal as the most influential candidate markers for differentiating ripening stages. Firmicutes remained the dominant bacterial phylum. At the genus level, the community shifted from the initial predominance of *Brochothrix* toward enrichment of *Staphylococcus* and *Macrococcus*. Spearman correlation analysis revealed that dehydration, salt accumulation, and lipid oxidation were associated with the dominant species and various volatile aldehydes, esters, and alcohols. Overall, these findings clarify the multidimensional succession of quality and flavor during JR ripening and provide a basis for monitoring the critical ripening period and standardizing production.

## 1. Introduction

Jiangrou is a traditional meat product widely consumed in China because of its characteristic color, rich aroma, and complex taste [1,2]. Sichuan Jiangrou is a representative regional meat product from Southwest China. Its production involves sauce-based curing, flavor development from seasonings, and natural drying and ripening under relatively mild environmental conditions. Dry-cured hams mainly rely on prolonged salt curing and endogenous enzymatic reactions, whereas smoked bacon is characterized by smoking and dehydration. Fermented sausages are produced with starter cultures. In contrast, Sichuan Jiangrou undergoes sauce-based curing, seasoning, natural drying, and ripening under relatively mild conditions. These distinctive processing features lead to dynamic changes in salt diffusion, lipid oxidation, flavor-related compound transformation, and indigenous microbial succession during ripening [3,4]. Therefore, as a representative regional meat product, Sichuan Jiangrou has practical value for improving process control, quality evaluation, and the standardization of traditional meat production.

Recent research has focused on fermented and dry-cured meat products, including dry-cured ham, Chinese bacon, and fermented sausage, with particular emphasis on protein degradation, lipid oxidation, taste-active substances, volatile compounds, and microbial communities [5,6,7]. Proteolysis and lipolysis are two major biochemical processes involved in texture and flavor development in dry-cured meat products. They release peptides, free amino acids, and free fatty acids that serve directly or indirectly as flavor precursors [3]. In non-smoked Chinese bacon, hexanal and 1-octen-3-ol have been identified as important aroma contributors. The contents of key odorants differ among products from Sichuan, Chongqing, and Yunnan, highlighting the influence of regional processing conditions on aroma formation [8]. Studies on Longxi bacon have further revealed dynamic relationships among water activity, salt content, microbial succession, fatty acid transformation, and volatile compounds during ripening [9].

Microorganisms are another important driver of quality formation in naturally fermented meat products. Bacteria, yeasts, and molds originating from the raw materials, processing equipment, seasonings, and surrounding environment can participate in carbohydrate metabolism, proteolysis, lipolysis, amino acid catabolism, *β*-oxidation, and ester formation [10,11]. Frequently reported microorganisms in Chinese fermented meat products include staphylococci, lactic acid bacteria, micrococci, yeasts, and filamentous fungi, although their relative importance varies considerably among product types and processing regions [12]. In Suan zuo rou, regional differences in *Weissella*, *Staphylococcus*, *Brochothrix*, *Kazachstania*, and *Debaryomyces* were associated with variations in physicochemical properties and volatile compounds [13]. Similarly, dynamic microbial succession during Chinese fermented sausage ripening was accompanied by changes in amino acid, lipid, and carbohydrate metabolic pathways, with *Staphylococcus* and *Leuconostoc* showing close associations with flavor compounds [14]. These findings indicate that fermented meat quality is governed by the coordinated effects of moisture migration, salt diffusion, endogenous enzymatic reactions, oxidation, and microbial metabolism rather than by a single biochemical pathway. Nevertheless, published research has largely focused on finished products or on other categories of fermented and dry-cured meats. Although the general quality and flavor characteristics of Jiangrou are already recognized, the dynamic changes occurring throughout the ripening process remain insufficiently characterized. In particular, it remains unclear how dehydration, salt penetration, lipid oxidation, taste development, volatile aroma formation, and bacterial succession interact throughout ripening. This lack of systematic information makes it difficult to identify critical ripening stages and potential markers for process monitoring and quality control.

To address this gap, the present study systematically investigated the dynamic changes in the quality and flavor of Sichuan Jiangrou during ripening. Based on previous studies of fermented and dry-cured meat products, we hypothesized that progressive dehydration and salt accumulation during ripening would be associated with dynamic changes in physicochemical properties, flavor-related compounds, and bacterial community succession. We further hypothesized that stage-dependent variations in aroma-active compounds could serve as potential markers for distinguishing different ripening stages. Moisture and salt contents were measured to characterize dehydration and salt penetration. Peroxide value, thiobarbituric acid reactive substances (TBARS) content, and superoxide dismutase (SOD) activity were determined to evaluate lipid oxidation-related changes. Gas chromatography-mass spectrometry (GC-MS) was used to characterize volatile compounds, while electronic tongue analysis was performed to evaluate the overall taste profile. Free amino acids were further quantified to determine their accumulation patterns and potential contributions to taste formation. In addition, 16S rRNA gene sequencing was performed to characterize bacterial community succession during ripening. Finally, a machine learning model was employed to screen and rank aroma-active compounds, thereby identifying key discriminative aroma-active compounds associated with different ripening stages. This study aimed to clarify the dynamic evolution of quality and flavor in Sichuan Jiangrou and to provide a basis for ripening control, flavor improvement, and standardized production.

## 2. Materials and Methods

### 2.1. Preparation of Samples

Sichuan Jiangrou (JR) was prepared using fresh pork belly trimmed of surface fascia and cut into uniform strips (approximately 20 × 5 cm, 500 ± 10 g). The curing mixture contained soy sauce (15.00% of meat weight), sweet bean sauce (10.00%), salt (3.00%), and sugar (2.50%). It also contained liquor (4.00% *v*/*w*) and spices. The spices included star anise (0.75 g), cinnamon (0.50 g), Sichuan pepper (0.50 g), clove (0.25 g), and ginger powder (0.50 g). The meat strips were thoroughly mixed with the curing ingredients and subjected to ultrasound-assisted curing followed by vacuum tumbling. Ultrasound treatment was performed using an SB-5200DT (Ningbo Scientz Biotechnology Co., Ltd., Ningbo, China) at a frequency of 40 kHz and a power of 200 W for 30 min at 25 °C. The samples were subsequently vacuum-tumbled using a GR-20 (Shanghai Gaofu Machinery Equipment Co., Ltd., Shanghai, China) at a vacuum level of −0.08 MPa and a rotation speed of 15 rpm for 40 min at 4 °C. The curing process was then carried out in a climatic chamber at 4 °C for 7 days, with the samples manually turned every 24 h to ensure uniformity. Following curing, each sample was rinsed once with 1500 mL of water at 25 °C for 2 min. This corresponded to a water-to-meat ratio of 3:1 (*v*/*w*). After rinsing, excess water was removed by placing the samples on a clean mesh for 30 min, followed by air drying at 20 ± 1 °C and 55–60% RH for 12 h. During ripening, the meat was suspended in a climatic chamber maintained at 10.5 ± 0.5 °C. Relative humidity was set at 65% and controlled within ±3% RH. Temperature and relative humidity were monitored daily throughout the ripening period. A constant-temperature-humidity incubator (Model LHS-100CL, Shanghai Yiheng Scientific Instrument Co., Ltd., Shanghai, China) was used to simulate the typical winter microclimate of Chengdu, Sichuan. Based on preliminary experiments, the ripening period was set at 35 days. During the ripening period, samples were collected at five-day intervals (JR0, JR5, JR10, JR15, JR20, JR25, JR30, and JR35). The collected samples were immediately vacuum-packed and stored at −18 °C for subsequent analysis.

### 2.2. Determination of Moisture and Salt Contents

The moisture content was determined using the direct drying method described by Xu et al. [15]. Briefly, approximately 5.00 g of homogenized sample was placed in a pre-weighed aluminum dish. The sample was dried in a forced-air oven at 105 ± 2 °C to constant weight. The moisture content was calculated as the percentage of weight loss relative to the initial sample weight using the following equation:

(1) Moisture content (%) = (W_2_ − W_1_)/W_3_ × 100

where W_1_ is the combined weight of the aluminum dish and sample after drying (g), W_2_ is the combined weight of the aluminum dish and sample before drying (g), and W_3_ is the initial weight of the sample (g).

Salt content was determined by potentiometric titration according to GB 5009.44-2016 [16]. Briefly, 5.0 g of homogenized sample was extracted with distilled water, filtered, and diluted to a fixed volume. An aliquot of the extract was acidified with nitric acid and titrated with standardized 0.100 mol L^−1^ AgNO_3_. The endpoint was determined from the inflection point of the potential-volume curve. Salt content was calculated from the AgNO_3_ consumption after blank correction and expressed as g NaCl per 100 g of sample on a wet-weight basis.

### 2.3. Determination of Peroxide Value (PV) and Thiobarbituric Acid Reactive Substances (TBARS) Value

Primary lipid oxidation was evaluated by determining the PV according to GB 5009.227-2023 [17]. Briefly, lipids were extracted from the homogenized samples and dissolved in a chloroform-acetic acid solution. After the addition of saturated potassium iodide, the liberated iodine was titrated with standardized sodium thiosulfate using starch as the indicator. PV was calculated after blank correction and expressed as g/100 g sample (peroxide equivalent to iodine). Secondary lipid oxidation was evaluated using the TBARS assay, with minor modifications to the method described by Chu et al. [18]. TBARS was used as a non-specific index of thiobarbituric acid-reactive secondary lipid oxidation products. Briefly, 5.00 g of the JR sample was homogenized with 20 mL of 7.50% (*w*/*v*) trichloroacetic acid containing 0.1% (*w*/*v*) EDTA. The homogenate was filtered, and 5.00 mL of the filtrate was mixed with an equal volume of 0.02 mol/L thiobarbituric acid solution. The mixture was heated at 95–100 °C for 30 min and cooled to room temperature. Absorbance was measured at 532 nm using a UV-Vis spectrophotometer (Shimadzu, Kyoto, Japan). A calibration curve was prepared using 1,1,3,3-tetraethoxypropane. Results were expressed as mg MDA equivalents/kg meat.

### 2.4. Determination of Superoxide Dismutase (SOD)

SOD activity was determined according to the Chinese national standard method (GB/T 5009.171-2003 [19]). Briefly, the JR sample was added to a Tris-HCl buffer solution (pH 8.2), and the reaction was initiated by the addition of pyrogallol. The absorbance of the reaction mixture was monitored spectrophotometrically at 325 nm. One unit of SOD activity (U) was defined as the amount of enzyme required to inhibit the autoxidation rate of pyrogallol by 50% per min under the specified assay conditions. SOD activity was expressed as specific activity (U/mg protein). For normalization, the soluble protein content of the corresponding extract was determined using a BCA protein assay with bovine serum albumin (BSA) as the standard. Absorbance was measured at 562 nm, and protein concentration was calculated from the BSA calibration curve. Specific SOD activity was calculated by dividing the measured SOD activity by the protein concentration of the corresponding extract. It was expressed as U/mg protein.

### 2.5. E-Tongue Evaluation

The instrumental taste-profile evaluation of JR was conducted using an electronic sensing system (Alpha MOS, Toulouse, France). Non-volatile taste-related response patterns were analyzed using an α-ASTREE electronic tongue equipped with a cross-selective sensor array. The array included sensors associated with sourness (AHS), saltiness (CTS), umami (NMS), bitterness (SCS), and sweetness (ANS). Before analysis, the sensor array was conditioned and calibrated according to the manufacturer’s standard procedures. Calibration was performed using the manufacturer-specified standard reference solutions to establish the sensor responses for the corresponding taste attributes. Sensor stability was checked before the analytical sequence. Between consecutive measurements, the sensors were rinsed with deionized water for 30 s to minimize carry-over effects. Sample preparation involved extracting 20.00 g of homogenized JR sample with 100.00 mL of deionized water for 10 min [20]. After filtration to remove lipids and insoluble particles, 80.00 mL of the aqueous extract was transferred to the measurement vessel. The samples were analyzed in random order, and each sample was measured in triplicate. Each measurement lasted 120 s with continuous stirring at 60 rpm, and the mean response recorded between 100 and 120 s was used as the output value.

### 2.6. Determination of Free Amino Acids (FAAs)

Free amino acids were quantified according to Wang et al., with minor modifications [21]. Briefly, 5.00 g of homogenized JR sample was mixed with 20.00 mL of 7% sulfosalicylic acid and subjected to ultrasonic extraction for 30 min. The extract was centrifuged at 10,000× *g* for 15 min. The resulting supernatant was passed through a 0.22 μm membrane filter. A 50.00 μL aliquot of the filtrate was injected into an automatic amino acid analyzer (SYKAM, Munich, Germany) fitted with an LCA K07/Li cation exchange column (150 × 4.60 mm). Amino acid derivatives were detected at 570 and 440 nm after post-column reaction with ninhydrin. During analysis, the column temperature was programmed from 20 to 99 °C. The reactor temperature was maintained at 130 °C, and the ninhydrin reagent was delivered at 0.25 mL/min. A mixed amino acid standard solution was used for compound identification and quantification. Individual amino acids were identified by comparing their retention times with those of authentic standards. They were quantified using external calibration curves established from the mixed amino acid standard solution. The detected amino acids were classified into umami, sweet, bitter, and tasteless groups according to previously reported taste properties [21].

### 2.7. Determination of VOCs by HS-SPME-GC-MS

To analyze the volatile compounds in JR samples, HS-SPME-GC-MS was performed according to Cheng et al. [22], with modifications. Briefly, 5.00 g of homogenized sample and 20.00 μL of 2-methyl-3-heptanone solution (5.00 μg/mL) were transferred into a 20 mL headspace vial. This compound served as the internal standard. The vial was immediately sealed with a polytetrafluoroethylene/silicone septum and equilibrated at 60 °C for 15 min. A preconditioned DVB/CAR/PDMS fiber was subsequently exposed to the vial headspace at 60 °C for 30 min. After extraction, the fiber was immediately inserted into the GC injector and thermally desorbed at 250 °C for 5 min. GC-MS analysis was performed using an SQ680 system equipped with an Elite-5MS capillary column (30 m × 0.25 mm × 0.25 μm; PerkinElmer, Waltham, MA, USA). Helium (≥99.99%) was used as the carrier gas at a constant flow rate of 1.00 mL/min. The oven temperature was initially maintained at 40 °C for 3 min, increased to 150 °C at 5 °C/min and held for 5 min, and then increased to 250 °C at 10 °C/min and held for 5 min. The mass spectrometer was operated in electron ionization mode at 70 eV, with ion-source and quadrupole temperatures of 230 and 150 °C, respectively. Mass spectra were acquired in full-scan mode over an m/z range of 20–400. For RI determination, a C8–C20 n-alkane standard mixture (AccuStandard, New Haven, CT, USA) was analyzed using the same chromatographic program as that applied to the samples. The retention times of the alkanes were recorded and subsequently used to calculate the linear retention indices of the volatile compounds as follows:

(2) $${RI}_{x}=100n+100\left[\frac{t_{R}(x)-t_{R}(n)}{t_{R}(n+1)-t_{R}(n)}\right]$$

Volatile compounds were identified by comparing their mass spectra with the NIST library, and only compounds with match scores above 800 were included in the analysis. Volatile compounds were semi-quantified relative to the internal standard according to Guo et al. [23], and the results were expressed as estimated concentrations (mg/kg).

### 2.8. Calculation of Estimated Odor Activity Values (OAVs)

To assess the potential contribution of volatile compounds in JR samples to the overall aroma profile, estimated OAVs were calculated using the semi-quantified concentrations of volatile compounds and odor threshold values reported in the literature. Because the odor thresholds used were reported in air rather than in meat, the calculated values were treated as estimated OAVs and were used only to screen potentially aroma-active compounds [24].

(3) $${\mathrm{OAV}}_{i}=\frac{C_{i}}{T_{i}}$$

In this equation, *C_i_* and *T_i_* denote the compound’s concentration and its odor threshold in air, respectively. The source, threshold value, unit, and reference for each compound are provided in Supplementary Table S2.

### 2.9. Machine Learning Model Construction

Compounds with an OAV greater than 1 in at least one ripening stage were retained according to a predefined aroma activity criterion and subsequently used for machine learning analysis [25]. The OAV profiles of the retained compounds were used as predictor variables, whereas the ripening stages were treated as class labels. An XGBoost classifier was selected because its gradient-boosted decision tree framework can effectively capture nonlinear relationships and higher-order interactions among aroma compounds [26]. Moreover, its regularization and shrinkage enable control of model complexity. The machine learning analysis included 24 samples from eight ripening stages, with three biological replicates per stage. Before model construction, all predictor variables were standardized using the StandardScaler function. To prevent information leakage, the scaler was fitted to the training data within each cross-validation fold and then applied to the corresponding validation data. Model hyperparameters were optimized using randomized search with three-fold stratified cross-validation. Three-fold stratified cross-validation was performed once with a random seed of 42. The search included learning rate, tree depth, number of estimators, subsampling ratio, and feature-sampling ratio. The predictive performance of the model was assessed using accuracy, macro-averaged F1 score, Cohen’s kappa coefficient, and the Matthews correlation coefficient (MCC) [27]. For multiclass ROC analysis, a one-vs-rest strategy was used, and the overall AUC was calculated as the macro-averaged AUC across all ripening stages. Accuracy represented the overall proportion of correctly classified samples. The macro F1 score assigned equal weight to each ripening stage. It therefore provided a balanced assessment of precision and recall across all classes. Cohen’s kappa quantified the agreement between predicted and observed classifications after correcting for agreement expected by chance. MCC was calculated from the complete multiclass confusion matrix. It provided a comprehensive measure of classification quality that was relatively robust to differences in class distribution. Performance metrics were calculated for each cross-validation fold and summarized across the three folds. To improve model interpretability, SHapley Additive exPlanations (SHAP) analysis was performed on the optimized XGBoost model. SHAP values were calculated to quantify the magnitude of the contribution of each odor-active compound to the classification of individual samples. The global importance of each compound was determined by averaging the absolute SHAP values across all samples and ripening classes. Compounds were ranked in descending order based on their absolute SHAP values, and compounds with higher values were identified as more important markers for discriminating between different ripening stages. Machine learning and SHAP analyses were conducted in Python (version 3.14) using the scikit-learn, XGBoost, and SHAP libraries.

### 2.10. DNA Extraction and PCR Amplification

Genomic DNA was extracted from JR samples using the DNeasy PowerFood Microbial Kit (Qiagen, Hilden, Germany) according to the manufacturer’s instructions. The V3-V4 hypervariable region of the bacterial 16S rRNA gene was amplified using primers 338F (5′-ACTCCTACGGGAGGCAGCAG-3′) and 806R (5′-GGACTACHVGGGTWTCTAAT-3′). Paired-end sequencing (2 × 250 bp) was performed on an Illumina MiSeq platform (Illumina, San Diego, CA, USA). Raw reads were quality-filtered and processed using the DADA2 plugin in QIIME2 (version 2023.2). Chimeric sequences were removed, and amplicon sequence variants (ASVs) were generated. Taxonomic classification was assigned against the SILVA 138 reference database. Alpha diversity indices, sequencing coverage, and alpha diversity indices, including observed Sobs, ACE, Chao1, Shannon, and Simpson diversity index (1-D), were calculated to evaluate microbial richness and diversity. Beta diversity was evaluated using Bray-Curtis distances. Non-metric multidimensional scaling (NMDS) was performed to visualize differences in bacterial community structure among ripening stages.

### 2.11. Statistical Analyses

All experimental data were obtained from three independent replicates and expressed as mean ± standard deviation. One-way analysis of variance (ANOVA) was performed using R software (version 4.4.2), followed by multiple comparisons with Tukey’s HSD test. Differences were considered statistically significant at *p* < 0.05. Significance annotations were generated using the agricolae package. PCA was conducted using the original sensor response matrix without additional centering or scaling. It was used to explore taste-related response patterns among ripening stages. Sample distributions and key influencing variables were visualized using score and loading plots. The score plot included 95% confidence ellipses to illustrate the sample distribution within each ripening stage. Bar plots and radar charts were produced using Origin 2024 software (OriginLab, Northampton, MA, USA).

## 3. Results

### 3.1. Analysis of Moisture Content and Salt Content

**Figure 1:**
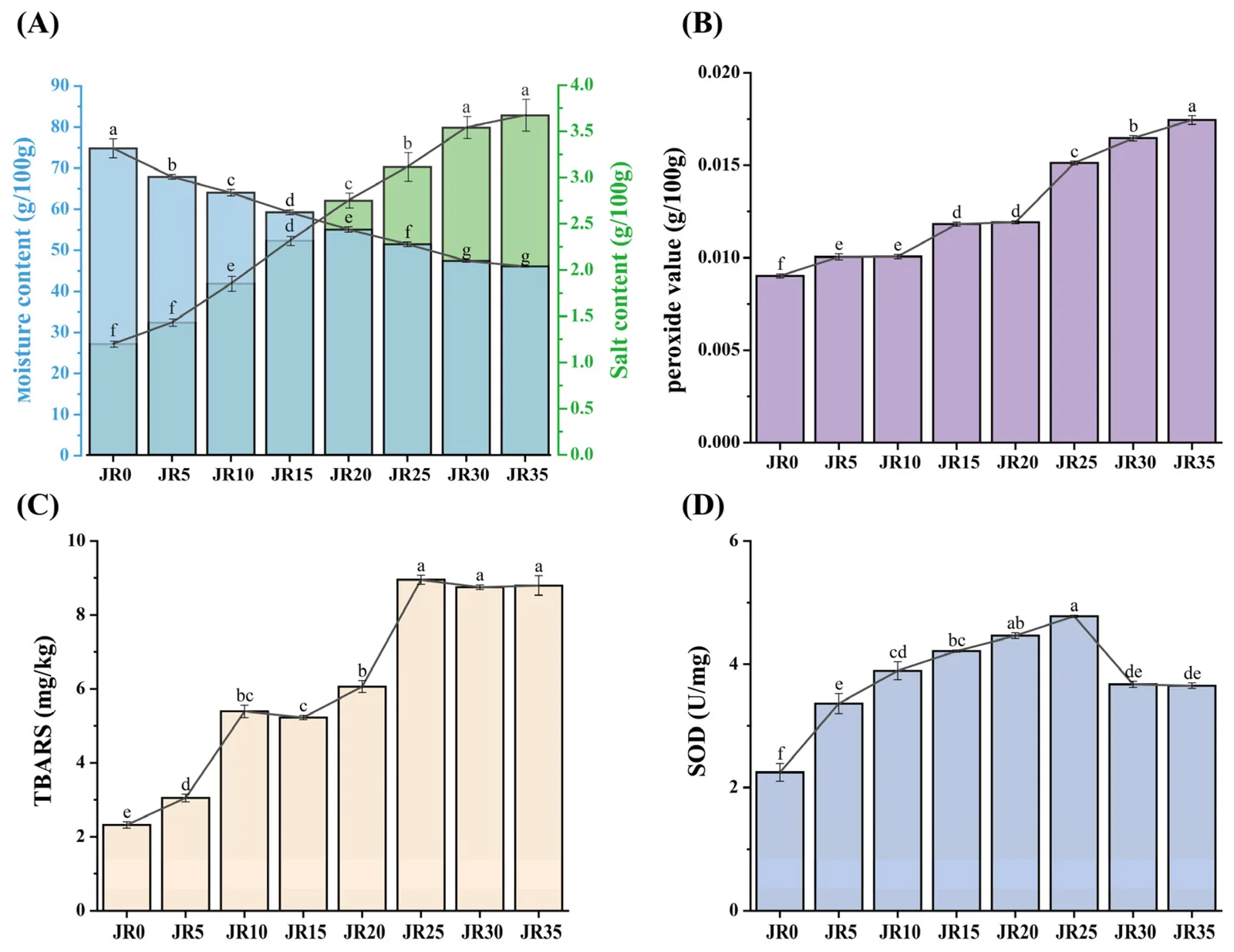
Changes in physicochemical properties during the ripening of JR. (**A**) Moisture and salt contents. (**B**) Peroxide value. (**C**) TBARS value. (**D**) SOD activity. Different lowercase letters indicate significant differences among ripening stages (*p* < 0.05).

Moisture and salt contents jointly reflected the dynamic changes in water migration, dehydration-driven concentration, and salt diffusion during the ripening of traditional Sichuan fermented sauced meat. As shown in Figure 1A, the moisture content decreased significantly from an initial value of 74.60 to 45.87 g/100 g during ripening (*p* < 0.05). Although moisture loss continued during the late ripening stage, the rate of decrease became slower, indicating that most dehydration occurred during the early and middle stages of ripening. This reduction was attributed not only to surface evaporation but also to the osmotic pressure gradient generated by the progressive penetration of salt into the muscle. As the salt concentration increased, free water and a portion of the weakly bound water migrated from the muscle tissue and subsequently evaporated, thereby accelerating sample dehydration [28]. Water loss also reduces the spacing between myofibrils and promotes muscle fiber shrinkage, contributing to the compact structure [10]. The reduced rate of moisture loss during the late ripening stage suggests that the major dehydration phase had largely occurred before this period under the present processing conditions. Thus, approximately day 25 may represent an important transition from rapid dehydration to a slower moisture-loss stage during ripening. In contrast to moisture content, salt content increased significantly throughout ripening (*p* < 0.05). The increase was particularly pronounced after JR10, and salt content reached a maximum of 3.67 g/100 g at JR35. This increase likely resulted from both the continuous diffusion of salt into the muscle tissue and the concentration effect associated with water loss. The relatively stable salt content during late ripening suggests that the rates of salt diffusion and water migration gradually became limited under the present conditions.

### 3.2. Analysis of Peroxide Value and TBARS

The PV and TBARS values were used to evaluate the accumulation of primary and secondary lipid oxidation products, respectively. As shown in Figure 1B,C, both PV and TBARS increased significantly with ripening time, indicating that lipid oxidation occurred throughout the ripening process of traditional Sichuan fermented sauced meat (*p* < 0.05). PV increased from 0.009 g/100 g in JR0 to 0.017 g/100 g in JR35, indicating the progressive formation and accumulation of primary oxidation products. Water loss can increase the concentrations of lipids and pro-oxidative components. Salt penetration destabilizes cellular membranes and promotes heme iron release, thereby facilitating free radical reactions and accelerating unsaturated fatty acid oxidation [29]. Consistent with the PV results, the TBARS value increased significantly from 2.32 mg/kg in JR0 to 8.95 mg/kg in JR25, with pronounced increases observed at JR10 and JR20. This pattern suggests that lipid hydroperoxides formed during early ripening progressively decomposed into secondary oxidation products, including aldehydes and ketones, during middle and late ripening [30]. Previous studies have demonstrated that moderate lipid oxidation may contribute to the generation of volatile compounds such as hexanal, heptanal, ketones, and alcohols, which provide fatty, meaty, and cooked aroma notes in fermented meat products [4]. Therefore, the increase in lipid oxidation observed in the present study may be closely associated with the development of the characteristic aroma of fermented sauced meat. However, excessive lipid oxidation may also generate rancid or otherwise undesirable odor notes. Accordingly, the increase in lipid oxidation observed in the present study was associated with changes in the volatile profile rather than necessarily indicating favorable aroma development. Notably, the TBARS values did not differ significantly between JR25 and JR35, whereas PV remained at a relatively high level (*p* > 0.05). This pattern may indicate that the formation and depletion of secondary oxidation products approached a relatively balanced state during late ripening. TBARS and other reactive carbonyl compounds do not necessarily accumulate continuously, as they may undergo further oxidation, volatilization, or reactions with proteins and amino compounds. Therefore, the stabilization of TBARS after JR25 does not indicate the termination of lipid oxidation but may reflect a balance between the generation and depletion of secondary oxidation products. Taken together, the changes in PV and TBARS suggest that JR20-JR25 may represent a period of intensified conversion of primary oxidation products into secondary products and associated changes in the volatile profile. After JR25, lipid oxidation appeared to enter a relatively stable stage. However, because trained sensory evaluation was not performed, whether these changes corresponded to desirable or undesirable sensory characteristics could not be directly determined.

### 3.3. Analysis of Superoxide Dismutase (SOD)

SOD is a key antioxidant enzyme that catalyzes superoxide anion radical dismutation, reducing oxidative stress and protecting cellular components from oxidative damage [31]. In post-mortem meat, however, measured SOD activity should be interpreted primarily as residual or extractable enzyme activity rather than as evidence of an active antioxidant response. As shown in Figure 1D, SOD activity in JR increased significantly during the early and middle stages of ripening, rising from 2.2 U/mg protein at JR0 to a maximum of 4.8 U/mg protein at JR25 (*p* < 0.05). The increase in measured SOD activity may reflect changes in the amount or extractability of residual SOD during processing. Because SOD activity was normalized to protein content, changes in protein concentration and solubility associated with moisture loss and proteolysis may also have affected the observed values. Therefore, the increase should not be interpreted as an active antioxidant response to oxidative stress. SOD activity then decreased markedly during late ripening, reaching approximately 3.6–3.7 U/mg protein at JR30 and JR35. These values were significantly lower than the value at JR25 (*p* < 0.05). This decrease may be associated with changes in enzyme stability and extractability as ripening progressed. Increasing salt concentration, moisture loss, proteolytic changes, and the accumulation of lipid oxidation products may alter protein structure or interfere with enzyme extraction and activity measurements.

### 3.4. Analysis of Electronic Tongue

**Figure 2:**
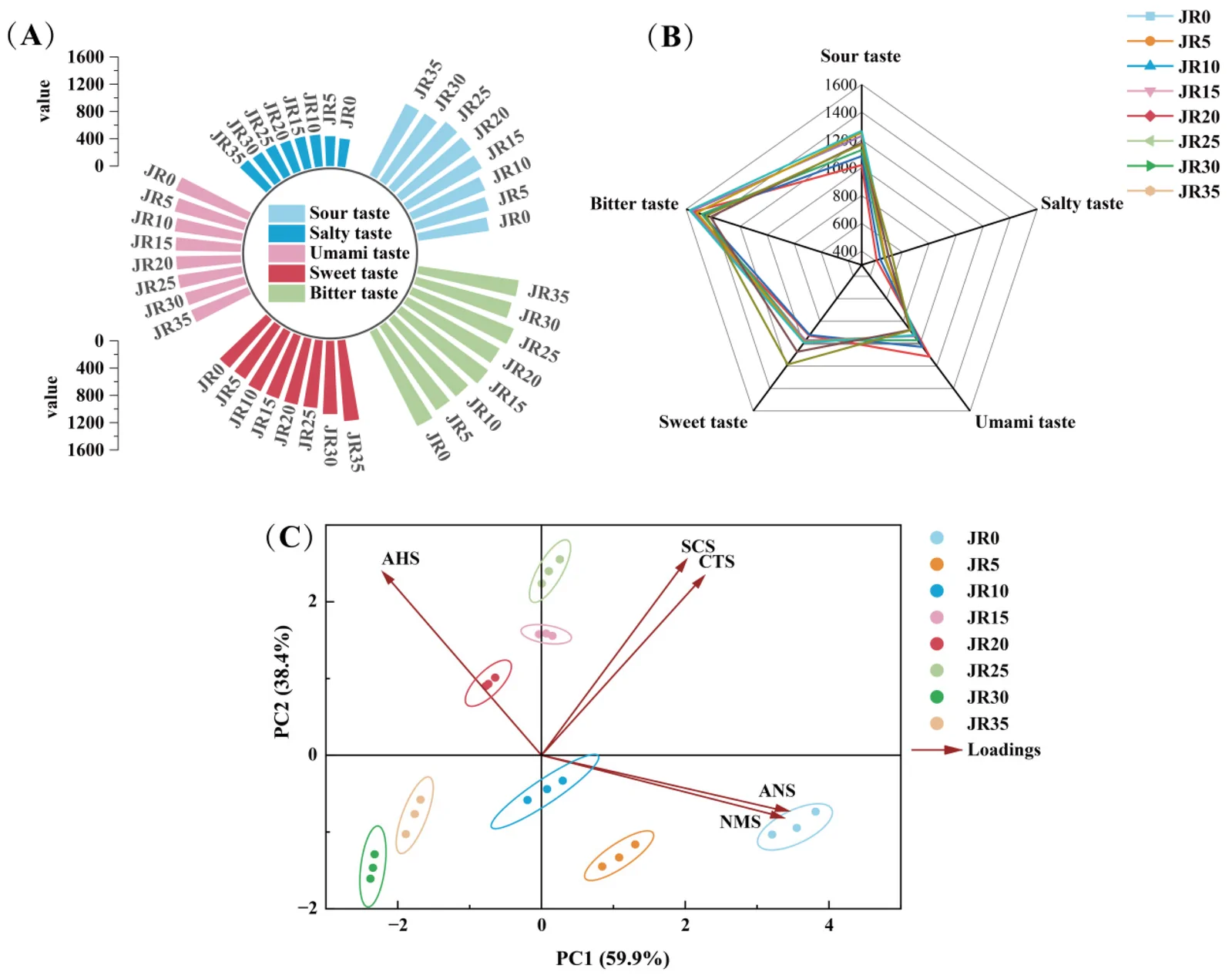
Electronic tongue analysis of JR during ripening. (**A**) Response intensities of five taste attributes. (**B**) Radar plots of taste profiles. (**C**) PCA score and loading plots.

The electronic tongue was used to objectively characterize changes in the overall taste profile of JR during ripening. This characterization was based on sensor responses associated with taste-related compounds [32]. The sensor responses of samples collected at different ripening stages are shown in Figure 2A,B. Clear differences were observed among the taste attributes throughout ripening, indicating that ripening substantially altered the taste characteristics of JR. Bitterness and sourness sensors exhibited relatively higher response values, whereas saltiness, umami, and sweetness sensors showed lower responses with varying fluctuations during ripening. These differences reflected distinct sensor response patterns associated with taste-related attributes throughout the ripening process. The sourness response value increased with ripening time. Changes in saltiness may be attributed to the continuous diffusion of salt into the meat tissue and the concentration effect resulting from moisture loss [21]. Variations in umami and sweetness were likely related to the release of free amino acids, small peptides, and other water-soluble taste-active compounds during protein degradation [33]. The variation in bitterness-related sensor response may be related to changes in taste-active compounds generated during protein degradation and lipid oxidation [34]. PCA further revealed differences in the taste profiles among ripening stages. As shown in Figure 2C, PC1 and PC2 explained 59.9% and 38.4% of the total variance, respectively, accounting for a cumulative contribution of 98.3%. This result indicates that the first two principal components adequately represented the overall variation in taste characteristics. Samples collected at different ripening times were clearly separated in the PCA score plot, demonstrating that the electronic tongue effectively distinguished the taste profiles of JR at different ripening stages. JR0, JR5, and JR10 were mainly distributed in the positive region of PC1 or close to the origin, indicating relatively similar taste characteristics during the early ripening stage. JR15, JR20, and JR25 shifted toward the positive direction of PC2, suggesting a pronounced transformation of the taste profile during the middle stage. JR30 and JR35 were further separated along the negative direction of PC1, indicating the gradual development of a mature taste profile distinct from those of the early and middle stages. According to the loading distribution, SCS and CTS were mainly located in the positive regions of PC1 and PC2 and contributed strongly to the discrimination of samples from the middle ripening stage. ANS and NMS were positioned in the positive region of PC1 and the negative region of PC2 and were more closely associated with the early-stage samples. In contrast, AHS was located in the negative region of PC1 and the positive region of PC2 and contributed substantially to the differentiation of samples from the middle and late ripening stages. The distinct response patterns of these sensors indicate their potential for discriminating samples from different ripening stages.

### 3.5. Analysis of Free Amino Acids (FAAs)

FAAs are important taste compounds and flavor precursors in fermented meat products, and their temporal changes reflect protein breakdown and subsequent amino acid transformation [35]. As shown in Table 1, the total FAA content in JR initially increased and subsequently decreased during ripening. It increased from 31.85 mg/100 g in JR0 to a maximum of 313.98 mg/100 g in JR25, remained relatively high in JR30, and then decreased to 233.85 mg/100 g in JR35. The pronounced accumulation during early and middle ripening was likely associated with the continuous hydrolysis of muscle proteins and peptides by endogenous and microbial proteolytic enzymes. In contrast, the late-stage decrease reflects the further utilization of FAAs through microbial metabolism, which can generate volatile aroma compounds [18]. The umami-related FAAs were primarily aspartic acid and glutamic acid, whose combined content increased from 4.85 mg/100 g in JR0 to 67.73 mg/100 g in JR30. In particular, glutamic acid increased from 3.37 to 42.30 mg/100 g and represented the principal umami-related FAA, while aspartic acid remained at relatively high levels between JR20 and JR30. The accumulation of these amino acids may have enhanced the umami characteristics of the samples during late ripening. The sweet-tasting FAA fraction, comprising threonine, serine, glycine, alanine, and proline, increased from 11.42 mg/100 g in JR0 to 106.39 mg/100 g in JR25 before subsequently declining. Alanine and proline were the predominant components of this fraction. Proline reached its maximum concentration in JR15, whereas alanine remained relatively abundant between JR25 and JR30. These changes suggest that sweet-tasting FAAs have contributed to a milder and more balanced taste profile during the middle and late stages of ripening. Bitter-tasting FAAs accounted for a substantial proportion of the total FAA pool. Their combined content increased significantly from 15.11 mg/100 g in JR0 to 142.03 mg/100 g in JR25 and then decreased slightly (*p* < 0.05). Histidine, leucine, lysine, valine, and arginine were the major components of this fraction. Their accumulation was consistent with extensive protein and peptide hydrolysis and may have contributed to the bitterness-related electronic tongue response. However, the actual sensory impact of these amino acids depends on their taste thresholds. A high concentration of bitter-tasting FAAs does not necessarily result in pronounced sensory bitterness. Their perception can be masked or modulated by saltiness, sweetness, umami, lipids, and aroma compounds [36]. Thus, the agreement between the FAA and electronic tongue results should be interpreted as supporting evidence. Cystine and methionine, which were grouped into the tasteless fraction in the classification used in this study, were detected at relatively low concentrations. Despite its limited abundance, methionine serves as a precursor for sulfur-containing volatile compounds through microbial catabolism and Strecker-related reactions. It thereby contributes to the overall aroma [33].

### 3.6. Analysis of Volatile Components

**Figure 3:**
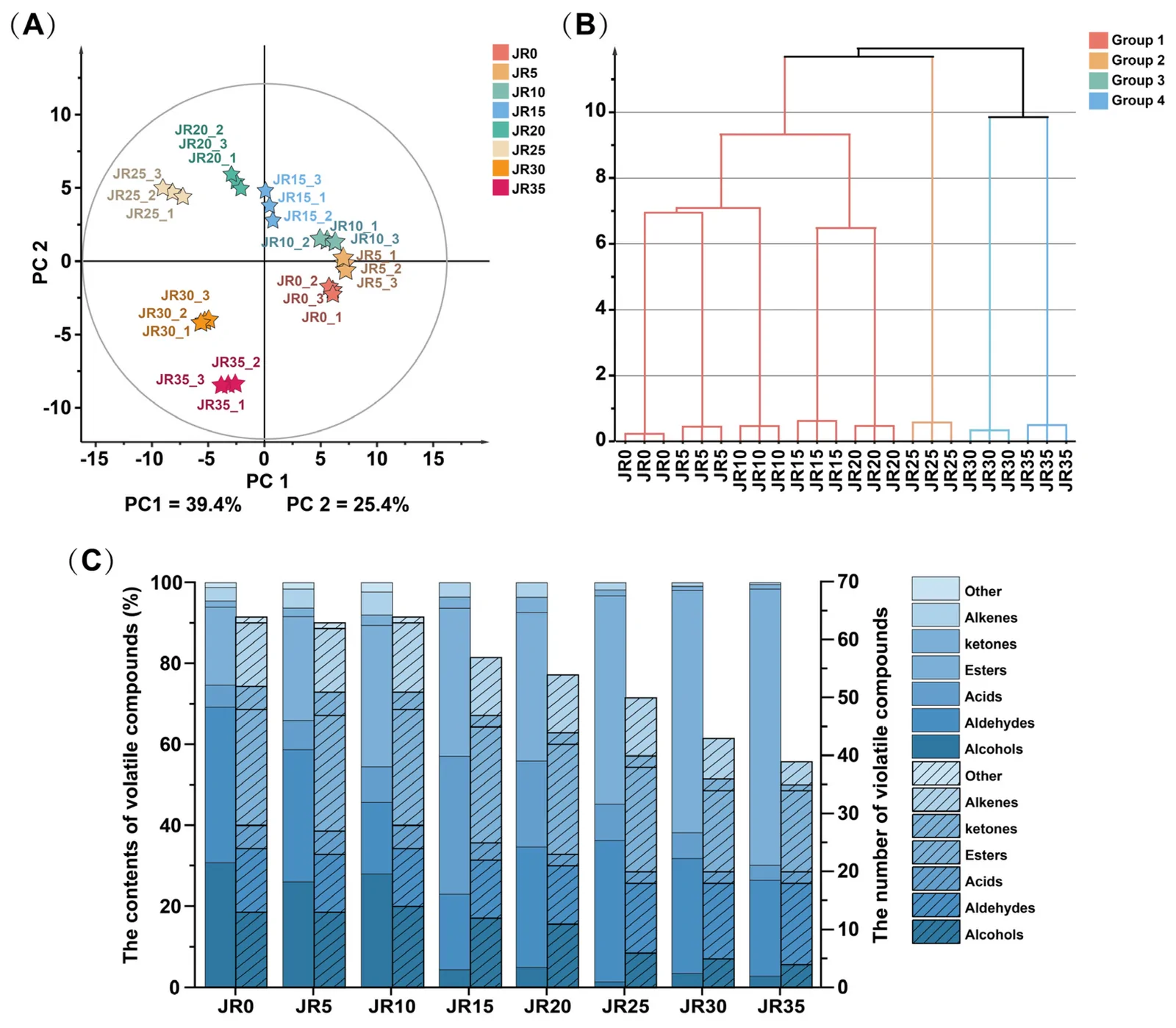
Changes in the volatile compound profiles of JR during ripening. (**A**) PCA score plot based on volatile compounds. (**B**) Hierarchical clustering analysis of volatile compound profiles. (**C**) Content of different classes of volatile compounds across ripening stages.

The volatile compounds of JR were identified using GC-MS, and the results are presented in Figure 3 and Table S1. In the PCA results, PC1 and PC2 explained 39.4% and 25.4% of the total variance, respectively, accounting for a cumulative contribution of 64.8% (Figure 3A). The score plot showed marked differences in volatile profiles among ripening stages, with JR35 exhibiting the greatest separation from the early-stage samples. These results indicate that the evolution of volatile compounds was strongly dependent on the ripening stage. Hierarchical clustering analysis (HCA) further supported and complemented the PCA results (Figure 3B). According to the dendrogram, JR0-JR20 formed a major cluster, whereas JR25, JR30, and JR35 were assigned to three separate clusters. In the major cluster, JR0 was most closely associated with JR5, and JR15 with JR20, indicating similar volatile profiles between adjacent stages. The independent clustering of JR25 and the subsequent separation of JR30 and JR35 revealed pronounced restructuring of the volatile profile after JR20. JR25 represented a potential transitional stage. Although JR20 and JR25 were positioned relatively close to each other in the PCA score plot, their separation in the HCA reflected differences distributed across variables not fully represented by PC1 and PC2. These remaining dimensions collectively accounted for 35.2% of the total variance.

Seven classes of volatile compounds were detected in JR, among which alcohols, aldehydes, acids, and esters were the predominant volatile classes (Figure 3C). Esters represented the most abundant volatile class during late ripening, with the highest quantified concentration among volatile classes observed in JR35 (933.18 mg/kg). This result was consistent with previous studies identifying alcohols, aldehydes, acids, and esters as major volatile classes in fermented meat products [37]. Similar volatile profiles have been reported in Chinese dry-cured ham and fermented sausages, in which aldehydes, alcohols, acids, and esters constitute the major aroma-related compound classes during ripening [10]. Esters can be generated through reactions between alcohols and carboxylic acids, which may be facilitated by microbial esterases [38]. Because many low-molecular-weight esters impart fruity, sweet, and floral notes, their pronounced accumulation in JR35 may contribute to the characteristic aroma of mature fermented JR [2]. However, the sensory importance of individual esters depends on their odor thresholds rather than solely on their concentrations. The concentration of alcohols reached a maximum value of 132.30 mg/kg in JR10, decreased sharply to 29.60 mg/kg in JR15, and remained relatively low thereafter. Alcohols in fermented meat originate from amino acid metabolism, carbohydrate fermentation, and the reduction of aldehydes, while also serving as substrates for ester formation [39]. One possible explanation is that the sauces and some seasoning ingredients used in JR formulas contain a relatively high amount of starch. During the ripening process, starch can be hydrolyzed into fermentable sugars by endogenous enzymes or microbial amylase, followed by the production of ethanol through glycolysis and alcohol fermentation pathways [30]. In addition, alcohols can also be derived from amino acid catabolism and the reduction of corresponding aldehydes [40]. Alcohols not only directly impart wine and ripening aromas to products but also serve as important precursors for ester formation. They can undergo esterification with organic acids, facilitated by microbial esterases. These reactions generate esters with fruity and sweet aroma characteristics [22]. Therefore, their decline after JR10 may partly reflect conversion into esters and other downstream products. The acid content initially increased from 17.57 mg/kg in JR0 to a maximum of 224.31 mg/kg in JR15 and subsequently declined to 50.76 mg/kg in JR35. The early accumulation of acids may have resulted from microbial carbohydrate and amino acid metabolism as well as lipid hydrolysis. The concurrent decline in acids and increase in esters during late ripening are consistent with the possible utilization of organic acids for ester formation. Aldehydes reached their highest concentration of 762.29 mg/kg in JR25. A similar accumulation of aldehydes during intermediate or late ripening has been observed in dry-cured ham and Chinese bacon, where lipid oxidation and amino acid degradation are considered important sources of these compounds [9]. In ripening sausages, however, aldehyde levels may decrease during prolonged ripening because of further reduction, oxidation, and microbial conversion, indicating that the temporal pattern of aldehydes can differ among meat products [14]. In fermented meat products, aldehydes are primarily formed through lipid oxidation and the Strecker degradation of amino acids. The pronounced accumulation of aldehydes at JR25 was consistent with the elevated lipid oxidation indices and FAA availability during middle to late ripening. This accumulation partly explained the transitional volatile profile of JR25. Aldehydes commonly possess low odor thresholds and can provide fatty, grassy, malty, and meaty notes [41]. However, excessive accumulation may also generate undesirable oxidized odors. In comparison, ketones, alkenes, and other compounds accounted for relatively small proportions of the total volatile content. Furthermore, a total of 64, 63, 64, 57, 54, 50, 43, and 39 volatile compounds were detected in JR0, JR5, JR10, JR15, JR20, JR25, JR30, and JR35, respectively. The number of detected compounds generally decreased with ripening time. Together with the increasing dominance of esters and the transient accumulation of aldehydes, this pattern suggests that aroma development in JR was characterized primarily by the selective transformation of volatile compounds and enrichment of dominant aroma-related constituents.

### 3.7. Machine Learning Analysis of Volatile Compounds

**Figure 4:**
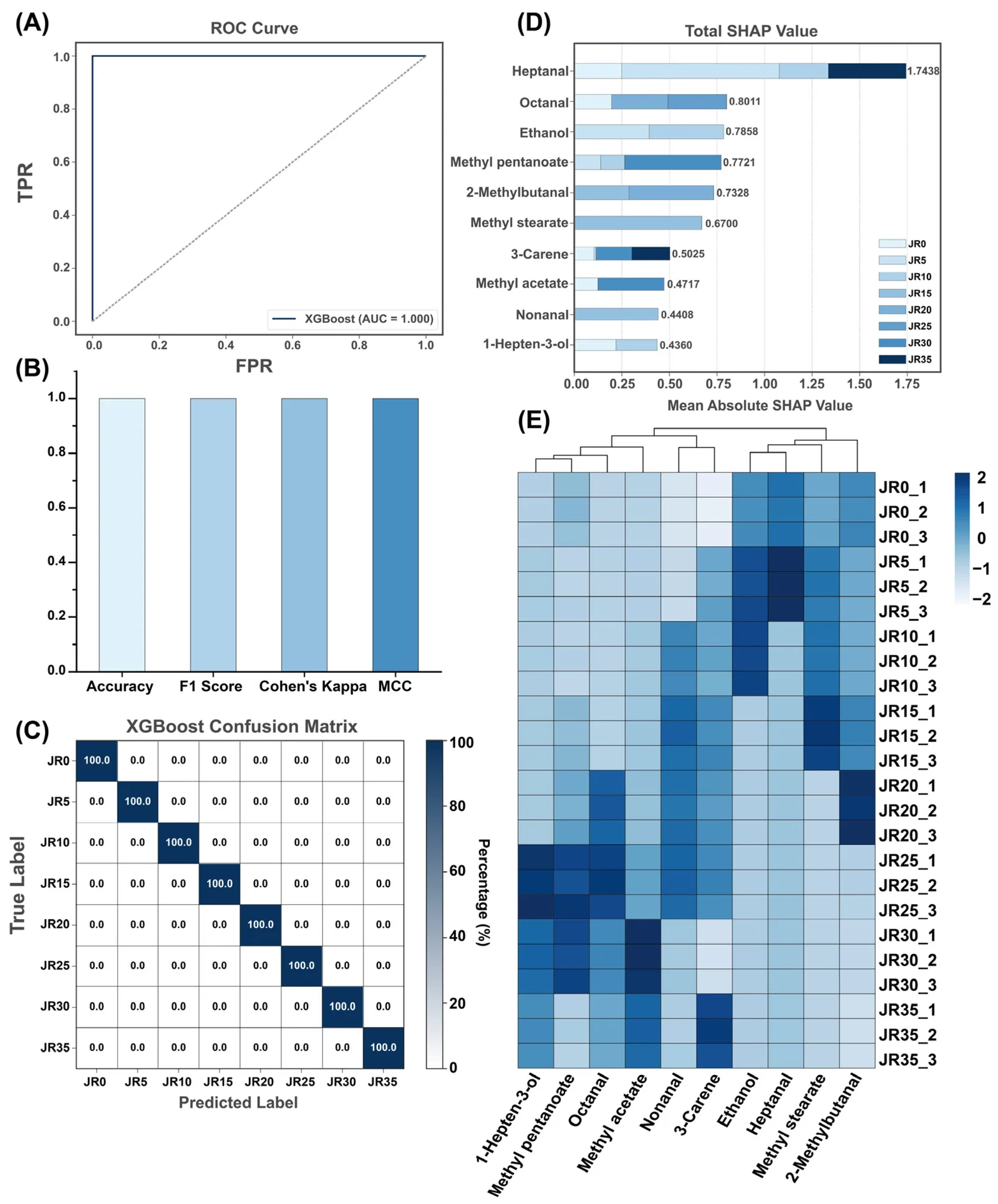
Screening of aroma-active compounds during JR ripening via the XGBoost model. (**A**) Receiver operating characteristic (ROC) curve. (**B**) Model performance evaluated by accuracy, macro F1 score, Cohen’s kappa coefficient, and Matthews correlation coefficient (MCC). (**C**) Normalized confusion matrix. (**D**) SHAP importance ranking of the top 10 aroma-active compounds. (**E**) Clustering heatmap of characteristic compounds.

To identify volatile markers associated with different ripening stages, an XGBoost classifier was constructed using aroma-active compounds with OAV greater than 1 (Table S2). The receiver operating characteristic curve yielded an AUC of 1.000 (Figure 4A). Similarly, the accuracy, F1 score, Cohen’s kappa coefficient, and MCC were all 1.000 (Figure 4B). The confusion matrix further showed that all samples were correctly assigned to their corresponding ripening stages, with no misclassification among JR0-JR35 (Figure 4C). These results are consistent with the stage-dependent separation observed in PCA and HCA, indicating that these aroma-active compounds contain information that distinguishes JR ripening stages. However, given the limited sample size, these results should not be interpreted as evidence of predictive robustness or generalizability. These findings are limited to the present dataset and require further validation using larger independent datasets. SHAP analysis was subsequently applied to quantify the contribution of each volatile compound to the model. Heptanal exhibited the highest mean absolute SHAP value (1.7438). It was therefore the most influential variable for differentiating JR ripening stages. (Figure 4D). It was followed by octanal (0.8011), ethanol (0.7858), methyl pentanoate (0.7721), 2-methylbutanal (0.7328), methyl stearate (0.6700), 3-carene (0.5025), methyl acetate (0.4717), nonanal (0.4408), and 1-hepten-3-ol (0.4360). The clustered heatmap further visualized the dynamic changes and clustering patterns of aroma-active compounds during ripening (Figure 4E). Ethanol and heptanal were enriched during early ripening, particularly in JR0-JR10, and generally decreased thereafter. Octanal was more prominent during the middle stage, especially in JR20-JR25. In contrast, 1-hepten-3-ol and methyl pentanoate were enriched mainly in JR25-JR30, whereas methyl acetate and 3-carene exhibited higher abundances in some late-stage samples. Methyl stearate and 2-methylbutanal also displayed stage-dependent enrichment patterns. Differences in these compounds provided the basis for the high classification performance of the XGBoost model. Among the ten selected compounds, aldehydes constituted the largest group and included heptanal, octanal, 2-methylbutanal, and nonanal. This result suggests that temporal variation in aldehydes contributed substantially to the discrimination among ripening stages. Heptanal, octanal, and nonanal are straight-chain aliphatic aldehydes that are mainly generated through the decomposition of lipid hydroperoxides formed during the oxidation of unsaturated fatty acids [35]. Due to their relatively low odor thresholds, these aldehydes can impart fatty, citrus-like, and meaty notes and contribute to mature meat aroma formation [5]. The stage distributions observed in this study were consistent with the changes in PV and TBARS, supporting an association between lipid oxidation and changes in the volatile profile. Lipid oxidation plays a critical role in the development of volatile compound profiles. In contrast, 2-methylbutanal is principally formed from isoleucine through microbial catabolism, or Strecker degradation, and is associated with malty, nutty, and roasted notes [42].

Ethanol showed a relatively high SHAP contribution among the alcohols. Previous studies have attributed ethanol in fermented meat products to carbohydrate metabolism by yeasts and other fermentative microorganisms and to the reduction of acetaldehyde [43]. In the present study, however, ethanol was more abundant during early ripening and subsequently decreased. One possible explanation is that part of the initial ethanol originated directly from sauces, liquor, or other seasoning ingredients used during curing. Its later decrease may have resulted from volatilization, oxidation, microbial utilization, or conversion into downstream products. Moreover, ethanol is an important precursor of ethyl esters and may indirectly promote fruity and ripening-related aroma formation [44]. The formation of 1-hepten-3-ol may also be associated with the secondary oxidation of unsaturated lipids. Its enrichment during JR25-JR30 was broadly consistent with the increases in PV and TBARS, suggesting that lipid-derived reactions continued during middle to late ripening. This compound has been reported to contribute fatty and buttery notes, with its sensory impact dependent on concentration and matrix [33]. Three esters were selected by the model, namely methyl acetate, methyl pentanoate, and methyl stearate. Methyl acetate and methyl pentanoate are relatively low-molecular-weight esters that may be formed through chemical or enzyme-mediated esterification of methanol with acetic and pentanoic acids, respectively [45]. In addition, their formation may also be facilitated by microbial esterase activity. Such short-chain esters generally exhibit fruity, sweet, and fresh odor characteristics and may reduce the sharp sensory impact of free organic acids [34]. Methyl pentanoate has previously been detected in fermented meat products, and its concentration changes according to the starter culture and ripening conditions [46]. In contrast, methyl stearate is a long-chain fatty acid methyl ester with a high molecular weight and low volatility [47]. Although this compound has been detected in fermented sausages and other processed meat products, its direct contribution to headspace aroma is hypothesized to be lower than that of short-chain esters. The monoterpene 3-carene was likely introduced through the spices used during JR processing rather than being produced directly by ripening microorganisms. Spices can markedly increase the abundance and diversity of terpenes in meat products, and 3-carene has been reported as an odor-active compound in seasoned meat products [48]. It is generally associated with herbal and slightly citrus-like notes and enhances the spicy character and aroma complexity of JR [49]. Its discriminatory importance across ripening stages reflects changes in its release from spices. However, given the limited sample size, these results should be interpreted cautiously and should not be considered evidence of predictive robustness or generalizability. These findings should be considered preliminary and require further validation using larger independent datasets.

### 3.8. Analysis of Microbial Communities

#### 3.8.1. Analysis of Microbial Diversity

**Figure 5:**
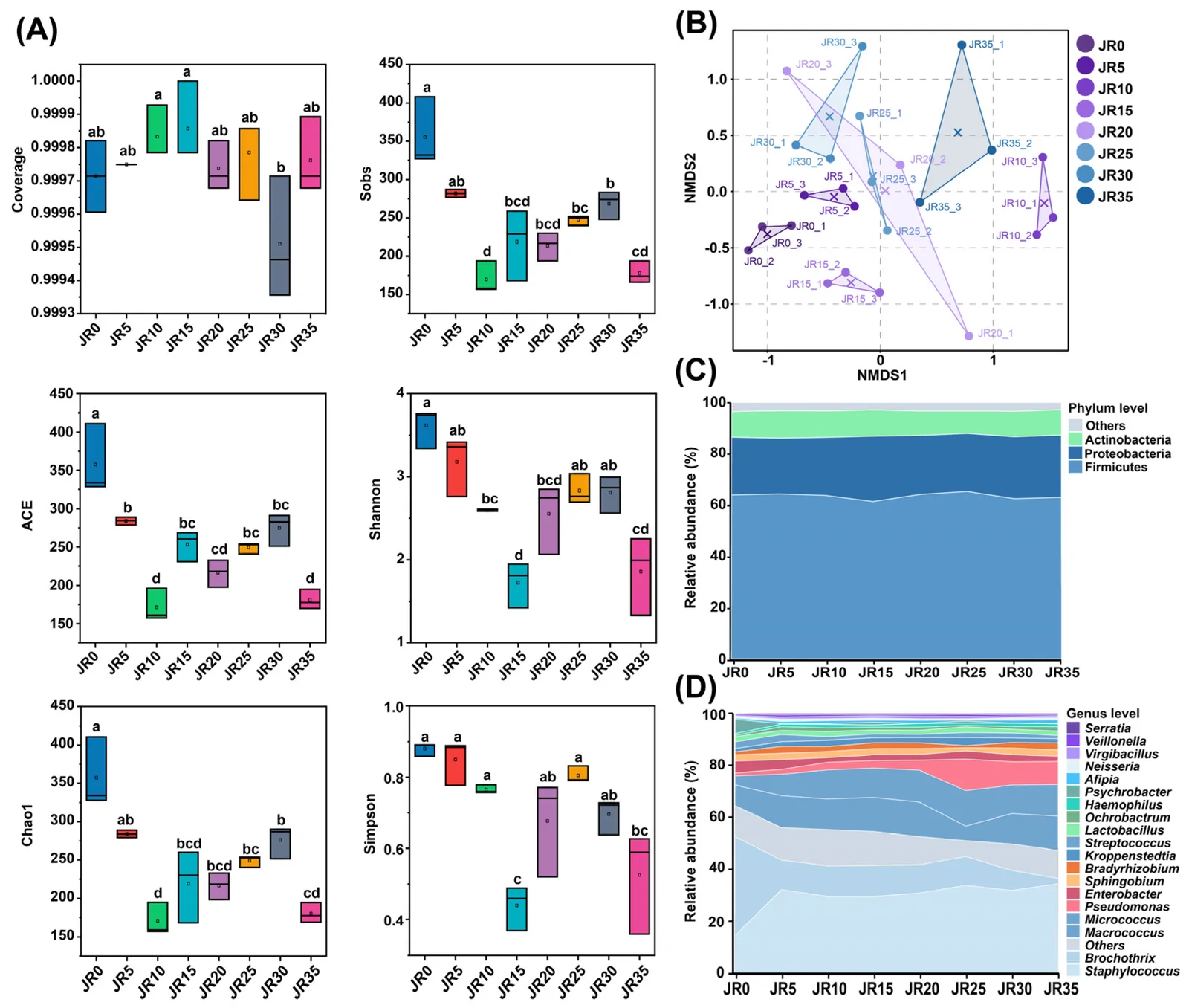
Bacterial diversity and community composition throughout the JR ripening process. (**A**) Alpha diversity indices of bacterial communities across distinct ripening stages, including Coverage, Sobs, ACE, Chao1, Shannon, and Simpson indices. (**B**) NMDS analysis of bacterial communities. (**C**) Bacterial taxa at the phylum level. (**D**) Bacterial taxa at the genus level. Note: Different lowercase letters above the bars indicate significant differences among ripening stages (one-way ANOVA followed by Tukey’s multiple comparison test, *p* < 0.05), whereas the same letters indicate no significant difference.

Alpha diversity analyses were performed to evaluate the richness, diversity, and structural variation of the bacterial community throughout JR ripening. The coverage values of all samples were consistently higher than 0.999, indicating that the sequencing depth was sufficient to capture the majority of bacterial taxa present in JR (Figure 5A). Nevertheless, bacterial richness and diversity varied markedly across ripening stages. The Sobs, ACE, and Chao1 indices decreased substantially from JR0 to JR10, partially recovered between JR15 and JR30, and subsequently declined again in JR35. Moreover, the richness indices of JR35 were significantly lower than those of JR0 (*p* < 0.05). These results indicate that ripening did not induce a simple linear reduction in bacterial richness but instead produced a stage-dependent restructuring of the bacterial community. A similar ripening pattern was observed for the Shannon index and Simpson diversity index (1-D). Both indices decreased during the early to middle ripening period, increased moderately from JR20 to JR30, and declined again at the end of ripening. Shannon and Simpson diversity indices (1-D) were significantly lower in JR35 than in JR0 (*p* < 0.05). This suggested that the final bacterial community exhibited reduced diversity and evenness. These environmental constraints further inhibit poorly adapted bacteria and favor the dominance of a few microbial groups adapted to ripening environments. These environmental constraints will further inhibit the growth of poorly adapted bacteria while making a few microbial groups that are adapted to ripening environments dominant [50]. Similar declines in microbial richness and diversity have also been reported during meat processing and maturation, owing to the progressive enrichment of microorganisms better adapted to the ripening environment. The NMDS analysis further revealed stage-associated differences in bacterial community structure (Figure 5B). Samples from the same ripening stage generally occupied similar regions, whereas early, intermediate, and late-stage samples showed varying degrees of separation. JR20 exhibited relatively large within-group dispersion, suggesting that this stage represented a period of pronounced and heterogeneous community restructuring. In contrast, the distributions of JR30 and JR35 differed from those of most early-stage samples, indicating that the bacterial community had developed toward a distinct late-ripening structure. Nevertheless, partial overlap among several adjacent stages showed that bacterial succession was gradual rather than completely discontinuous. This community transition was broadly consistent with the marked restructuring of the volatile profile around JR25. It suggests that changes in bacterial ecology accompany mature flavor profile formation.

#### 3.8.2. Analysis of Microbial Taxonomic Composition

Based on the sequencing results, bacterial community composition was further analyzed at the phylum and genus levels. The bacterial community structure showed distinct succession patterns during the ripening of JR. At the phylum level, Firmicutes, *Proteobacteria*, and *Actinobacteria* were identified as the predominant bacterial phyla throughout ripening (Figure 5C). Firmicutes consistently dominated the bacterial community, with abundances ranging from 61.35% to 64.40%, followed by *Proteobacteria* (21.60–25.47%) and Actinobacteria (9.43–10.69%). Other bacterial phyla accounted for only a minor proportion (<4%), indicating that ripening mainly reshaped the dominant bacterial groups rather than causing substantial changes in the overall phylum-level structure. The persistent predominance of Firmicutes is consistent with previous observations in traditional fermented meat products, including dry-cured ham and fermented sausages, where salt-tolerant Firmicutes are frequently enriched during maturation due to their strong adaptability to high osmotic pressure and reduced water activity [51,52,53]. In contrast, many environmental and raw-material-associated bacteria belonging to *Proteobacteria* are generally more sensitive to ripening stresses, resulting in fluctuations during maturation. In the present study, *Proteobacteria* increased moderately at JR15 and then declined slightly during late ripening. This pattern may reflect changes in oxygen availability, nutrient accessibility, and microbial competition. Although the abundance of major bacterial phyla remained stable, pronounced succession occurred at the genus level, suggesting that ripening primarily drove the replacement and enrichment of specific bacterial genera within dominant phyla.

At the genus level, a total of 19 dominant bacterial genera were identified during ripening (Figure 5D). The bacterial community exhibited clear succession characteristics, mainly characterized by the decline of *Brochothrix* and the enrichment of *Staphylococcus*, *Macrococcus*, and other salt-tolerant genera. At JR0, Brochothrix was the most abundant genus, accounting for 37.70% of the bacterial community, followed by *Staphylococcus* (14.77%), *Micrococcus* (7.91%), and *Psychrobacter* (5.40%). The high abundance of *Brochothrix* at the beginning of ripening is consistent with previous reports in fermented meat products, where *Brochothrix* is frequently detected in fresh meat and processing environments due to its adaptation to refrigerated storage and nutrient-rich meat matrices [54]. However, its abundance decreased continuously during ripening, reaching only 2.03% at JR35. This reduction may be attributed to increasing salt concentration, progressive dehydration, and competitive exclusion by more stress-tolerant microorganisms during maturation. In contrast, *Staphylococcus* rapidly increased from 14.77% at JR0 to 32.15% at JR5 and remained the predominant genus throughout ripening, reaching 34.53% at JR35. The enrichment of *Staphylococcus* is commonly observed in dry-cured and fermented meat products [12]. Coagulase-negative staphylococci possess strong tolerance to salt stress and low water activity and can survive under harsh curing conditions [55]. Previous studies have reported that coagulase-negative staphylococcal strains may possess proteolytic, lipolytic, and esterase activities. These activities may contribute to the release of free amino acids and fatty acids, which can serve as volatile aroma precursors during maturation [55,56]. Similarly, *Macrococcus* showed a gradual enrichment pattern, increasing from 3.60% at JR0 to 13.69% at JR25 and maintaining a high abundance at the end of ripening (12.08%). Previous studies have demonstrated that *Macrococcus* is commonly associated with fermented meat ecosystems and possesses strong environmental adaptability under curing conditions [57]. Its persistence during ripening suggests a possible association with microbial succession and flavor-related changes. In addition to Firmicutes-associated genera, several members of *Proteobacteria* exhibited stage-dependent changes. *Pseudomonas* showed a notable increase during the middle to late ripening period, particularly at JR25 (12.10%), compared with only 1.13% at JR0. Similar transient increases in *Pseudomonas* abundance have previously been reported in fermented meat products and are commonly associated with processing-related environmental conditions [18]. However, the increase in *Pseudomonas* at JR25 does not necessarily imply spoilage. It should be acknowledged that the present 16S rRNA sequencing analysis did not resolve *Pseudomonas* to the species level, and viable counts and direct spoilage and safety indicators were not measured. These limitations make it difficult to assess the specific quality and safety implications of the observed increase. The abundance of *Micrococcus* remained high during early and middle ripening stages (7.91–13.17%) but declined at JR25 before partially recovering at JR35. Members of *Micrococcus* have frequently been detected in cured meat products, and strains have been reported to be potentially associated with nitrate reduction, lipid degradation, and aroma development [58]. Meanwhile, other genera, including *Enterobacter*, *Sphingobium*, *Bradyrhizobium*, *Kroppenstedtia*, and *Lactobacillus*, were detected at low abundances and exhibited limited variations throughout ripening, suggesting that they may represent minor components of the microbial community during ripening.

### 3.9. Correlation Analysis of Physicochemical Properties, Microbial Genera, and Aroma-Active Compounds

**Figure 6:**
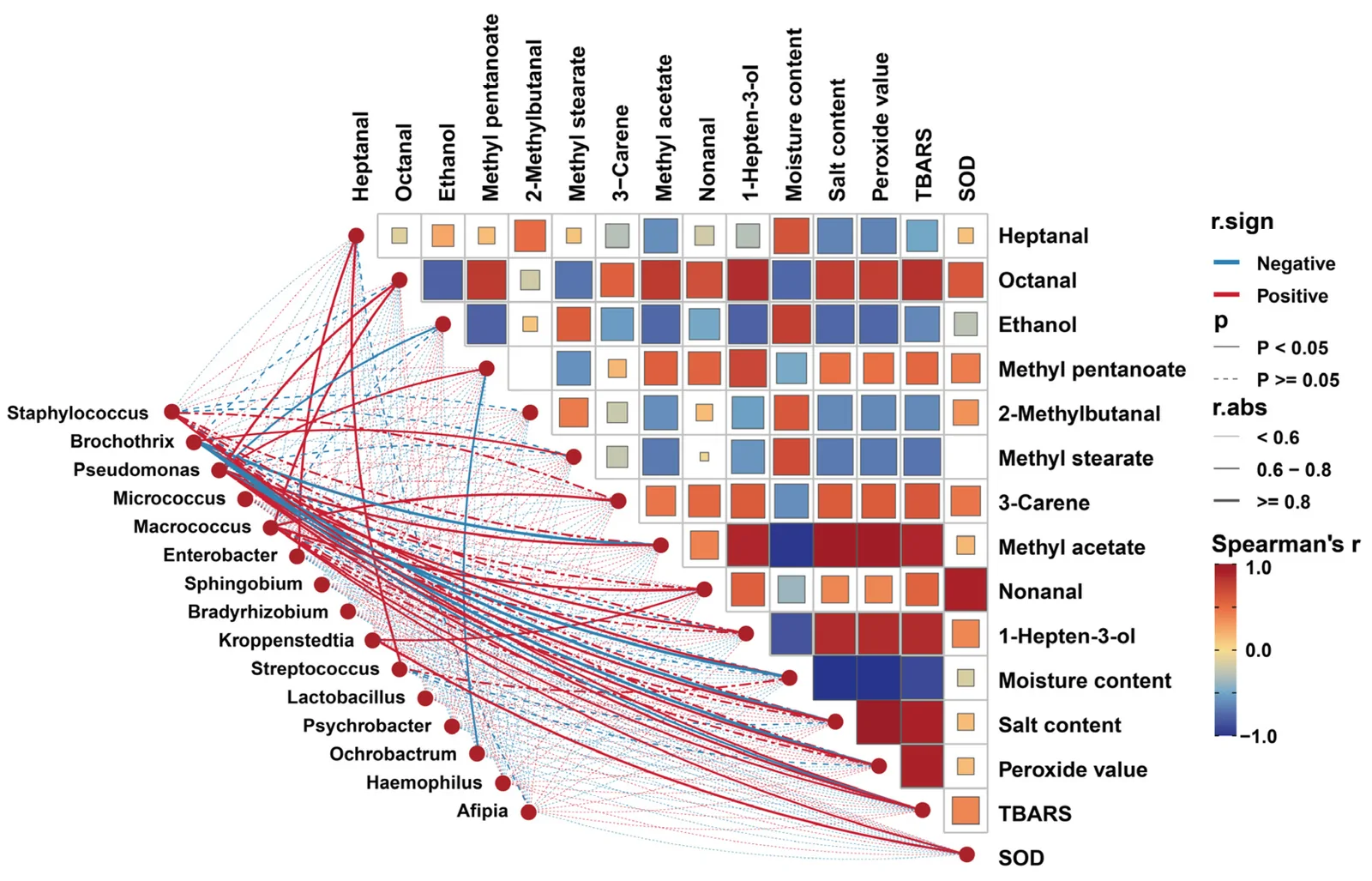
Spearman correlation analysis of bacterial genera, physicochemical properties, and aroma-active compounds during JR ripening.

Spearman correlation analysis was performed to further characterize the relationships among dominant bacterial genera, physicochemical properties, and aroma-active compounds during JR ripening (Figure 6). Moisture content was negatively correlated with salt content, peroxide value, and TBARS value, whereas these three maturation-related indices were positively correlated with one another. Octanal, methyl pentanoate, 3-carene, methyl acetate, nonanal, and 1-hepten-3-ol were generally positively correlated with these indices but negatively correlated with moisture content. Among them, methyl acetate, nonanal, and 1-hepten-3-ol showed particularly strong associations with physicochemical indicators that increased during the middle and late ripening stages. These results suggest that their accumulation was associated with dehydration, salt penetration, and lipid oxidation during ripening. Similar studies have identified moisture content, water activity, salt content, and pH as important factors associated with bacterial community structure and biochemical transformations during the maturation of cured meat products [59]. In contrast, heptanal, ethanol, 2-methylbutanal, and methyl stearate exhibited different correlation patterns. Heptanal was positively correlated with moisture content but negatively correlated with salt content, peroxide value, and TBARS value. Ethanol and methyl stearate also tended to be negatively associated with several physicochemical variables characteristic of later ripening stages. These contrasting patterns indicate that different aroma-active compounds were associated with distinct stages of JR ripening.

The genus variable network showed that *Staphylococcus*, *Brochothrix*, *Pseudomonas*, *Micrococcus*, and *Macrococcus* were highly connected with physicochemical indicators and aroma-active compounds, suggesting that these dominant genera were closely associated with the physicochemical and volatile changes occurring during ripening. In particular, the enrichment of Staphylococcus and *Macrococcus* during the middle and late stages coincided with changes in peroxide value, TBARS, and several aroma-active aldehydes and esters. Previous studies have reported that members of these genera may possess proteolytic, lipolytic, and esterase activities and may therefore be associated with protein and lipid degradation in fermented meat systems [53]. In studies of Longxi bacon, *Staphylococcus* and *Macrococcus* have also been proposed as potential contributors to protein and lipid degradation [9]. Moreover, previous studies have reported that strains of *Staphylococcus* carnosus possess *β*-oxidation and thioesterase activities that may be involved in the generation of lipid-derived aldehydes, including octanal [60]. Similar results were obtained in Chinese fermented sausage, where *Staphylococcus* was positively correlated with lipid and amino acid metabolism and with several differential volatile compounds [14]. The central position of Staphylococcus in this network is also consistent with observations in Panxian ham. In that product, Staphylococcus was identified as the dominant bacterium and was associated with amino acid and fatty acid production during ripening [61]. In contrast, *Brochothrix* was abundant at JR0 but declined thereafter, suggesting a stronger association with the initial meat matrix and high moisture conditions. High abundances of *Brochothrix* and Staphylococcus have also been reported in Suan zuo rou, where both genera were correlated with multiple volatile compounds [13]. The correlations involving *Pseudomonas*, *Psychrobacter*, *Enterobacter*, and other members of *Proteobacteria* may reflect the combined effects of oxygen availability, raw-material-derived microbiota, and changes in nutrient availability during ripening [62]. *Psychrobacter*, in particular, has been proposed to participate in lipid metabolism and amino acid hydrolysis in fermented meat ecosystems [9]. These associations may reflect microbial metabolic contributions or coordinated responses of microbial abundance and volatile profiles to ripening. However, some of these correlations may simply reflect similar changes over ripening time rather than direct biological interactions. Therefore, they should be interpreted as associations, and further validation is needed to confirm causal relationships.

## 4. Conclusions

This study investigated the dynamic changes in physicochemical properties, taste-related characteristics, volatile profiles, and bacterial community succession during Sichuan Jiangrou ripening. The results revealed coordinated changes in moisture loss, lipid oxidation, flavor compound transformation, and microbial succession during ripening. The period around days 20–25 represented an important transition stage in quality development. The integration of GC-MS, 16S rRNA sequencing, correlation analysis, and XGBoost-SHAP analysis identified key volatile compounds and microbial patterns associated with different ripening stages. These findings provide insights into changes in flavor and microbial characteristics during JR ripening and help explain process variation. However, these markers and microbial-associated substances require further verification using larger independent sample sets and functional experiments before they can be applied in production.

## Figures and Tables

**Table 1:** Free amino acid content of JR at different ripening stages (mg/100 g).

Flavor Characteristics	Amino Acids	Samples							
		JR0	JR5	JR10	JR15	JR20	JR25	JR30	JR35
Umami	Aspartic acid	1.48 ± 0.07 ^d^	12.60 ± 2.55 ^c^	13.09 ± 1.54 ^c^	19.91 ± 1.91 ^b^	26.46 ± 3.26 ^a^	26.27 ± 2.09 ^a^	25.43 ± 0.89 ^a^	15.82 ± 1.61 ^c^
	Glutamic acid	3.37 ± 0.30 ^d^	20.70 ± 3.28 ^c^	32.93 ± 6.33 ^b^	33.51 ± 3.77 ^b^	32.27 ± 5.30 ^b^	34.64 ± 5.22 ^b^	42.30 ± 3.07 ^a^	26.90 ± 2.29 ^bc^
Sweet	Threonine	0.88 ± 0.20 ^d^	4.78 ± 0.40 ^c^	12.66 ± 1.71 ^a^	9.31 ± 1.10 ^b^	12.63 ± 0.21 ^a^	12.65 ± 1.56 ^a^	10.93 ± 0.25 ^ab^	9.02 ± 1.50 ^b^
	Serine	1.35 ± 0.30 ^e^	7.04 ± 1.76 ^d^	6.97 ± 1.31 ^d^	15.83 ± 2.36 ^b^	17.05 ± 1.23 ^a^	18.57 ± 1.16 ^ab^	16.09 ± 1.50 ^ab^	12.74 ± 0.51 ^c^
	Glycine	0.99 ± 0.12 ^e^	5.95 ± 1.20 ^d^	12.09 ± 1.05 ^bc^	11.14 ± 0.55 ^bc^	13.26 ± 1.16 ^ab^	14.73 ± 2.94 ^a^	13.28 ± 0.87 ^ab^	10.61 ± 0.75 ^c^
	Alanine	1.88 ± 0.23 ^d^	11.64 ± 1.70 ^c^	20.92 ± 2.18 ^b^	23.22 ± 4.58 ^b^	25.74 ± 2.71 ^ab^	29.78 ± 3.46 ^a^	28.88 ± 3.88 ^a^	23.22 ± 2.04 ^b^
	Proline	6.32 ± 0.83 ^e^	24.63 ± 4.98 ^cd^	33.30 ± 3.25 ^b^	43.24 ± 5.04 ^a^	33.67 ± 6.87 ^b^	30.66 ± 2.14 ^bc^	30.47 ± 0.99 ^bc^	23.31 ± 3.08 ^d^
Bitter	Valine	1.35 ± 0.22 ^d^	7.51 ± 0.73 ^c^	13.66 ± 2.05 ^b^	15.36 ± 1.96 ^ab^	17.45 ± 0.75 ^a^	17.69 ± 1.25 ^a^	16.70 ± 1.64 ^a^	12.93 ± 2.20 ^b^
	Isoleucine	1.20 ± 0.30 ^e^	5.84 ± 0.11 ^d^	7.20 ± 1.21 ^cd^	9.37 ± 1.04 ^bc^	14.34 ± 1.48 ^a^	13.60 ± 2.86 ^a^	13.58 ± 1.20 ^a^	10.44 ± 0.77 ^b^
	Leucine	2.36 ± 0.46 ^f^	11.44 ± 2.24 ^e^	14.15 ± 2.31 ^de^	15.45 ± 1.54 ^cd^	21.29 ± 2.14 ^ab^	18.96 ± 2.08 ^bc^	24.13 ± 3.21 ^a^	14.50 ± 1.82 ^de^
	Tyrosine	0.79 ± 0.03 ^d^	2.74 ± 0.55 ^c^	3.39 ± 0.29 ^c^	4.23 ± 0.38 ^b^	5.94 ± 0.55 ^a^	6.33 ± 0.77 ^a^	6.15 ± 0.43 ^a^	4.51 ± 0.17 ^b^
	Phenylalanine	1.03 ± 0.12 ^f^	4.65 ± 0.30 ^e^	6.30 ± 0.31 ^d^	6.76 ± 1.11 ^cd^	8.89 ± 1.00 ^b^	9.17 ± 0.35 ^b^	11.74 ± 0.91 ^a^	7.90 ± 0.93 ^bc^
	Histidine	4.08 ± 0.20 ^e^	19.20 ± 2.74 ^d^	31.11 ± 0.67 ^bc^	31.06 ± 2.71 ^bc^	35.29 ± 4.32 ^ab^	39.94 ± 4.34 ^a^	23.20 ± 1.99 ^d^	28.78 ± 2.23 ^c^
	Lysine	2.53 ± 0.20 ^e^	9.84 ± 0.15 ^d^	14.04 ± 1.37 ^c^	19.95 ± 4.36 ^ab^	19.52 ± 1.25 ^ab^	21.63 ± 1.66 ^a^	20.17 ± 3.22 ^ab^	17.00 ± 1.96 ^bc^
	Arginine	1.77 ± 0.10 ^f^	6.22 ± 0.76 ^e^	10.23 ± 2.26 ^cd^	8.92 ± 1.32 ^de^	13.13 ± 0.57 ^bc^	14.71 ± 2.41 ^ab^	17.54 ± 3.20 ^a^	13.09 ± 0.31 ^bc^
Tasteless	Cystine	0.02 ± 0.00 ^d^	0.08 ± 0.01 ^c^	0.13 ± 0.02 ^b^	0.13 ± 0.02 ^b^	0.16 ± 0.02 ^b^	0.17 ± 0.02 ^b^	0.32 ± 0.04 ^a^	0.13 ± 0.02 ^b^
	Methionine	0.45 ± 0.10 ^e^	1.92 ± 0.20 ^d^	3.33 ± 0.10 ^c^	3.60 ± 0.60 ^bc^	4.49 ± 0.71 ^a^	4.48 ± 0.50 ^a^	4.31 ± 0.52 ^ab^	2.95 ± 0.20 ^c^

Different lowercase letters indicate significant differences among ripening stages (*p* < 0.05).

## Data Availability

The original contributions presented in this study are included in the article/Supplementary Material. Further inquiries can be directed to the corresponding authors.

## Supplementary Materials

The following supporting information can be downloaded at: https://www.mdpi.com/article/10.3390/foods15183258/s1, Supplementary Table S1. Volatile compounds identified in JR during ripening (mg/kg). Supplementary Table S2. OAVs of volatile compounds in JR samples during ripening.
