# Supplementary material for: Dynamic Changes in Flavor-Related Compounds and Bacterial Community Structure During Sichuan Jiangrou Ripening
Source: Foods. 2026 Sep 15;15(18):3258. doi: 10.3390/foods15183258 (PMC13606823; doi:10.3390/foods15183258)
Supplement: Supplementary file 1 [file foods-15-03258-s001.zip › foods-4519534-supplementary.pdf]

Supplementary Table S1. Volatile compounds identified in JR during ripening (mg/kg).

Class	Compounds	RT	RI	CAS	Formula	Sample						
						JR0	JR5	JR10	JR15	JR20	JR25	JR30
Alcohols	1-Hexanol	8.36	868	111-27-3	$C_6H_{14}O$	-	2.05	0.42	-	4.94	-	-
	3-Methyl-1-butanol	4.82	734	123-51-3	$C_5H_{12}O$	15.26	8.12	9.46	-	-	-	-
	2-Octyn-1-ol	8.09	1060	20739-58-6	$C_8H_{14}O$	0.81	-	-	-	-	-	-
	2-Ethyl-1-pentanol	15.02	902	27522-11-8	$C_7H_{16}O$	-	-	2.72	-	-	-	1.70
	1-Octen-3-ol	12.00	980	3391-86-4	$C_8H_{16}O$	-	-	-	-	2.89	-	-
	1-Hepten-4-ol	9.60	896	3521-91-3	$C_7H_{14}O$	3.06	3.02	-	-	-	1.53	-
	2-Ethylcyclobutanol	2.78	788	35301-43-0	$C_6H_{12}O$	-	-	-	-	23.83	-	-
	1-Hepten-3-ol	11.96	903	4938-52-7	$C_7H_{14}O$	-	0.56	2.43	12.27	-	2.06	7.12
	1,3-Propanediol	3.53	917	504-63-2	$C_3H_8O_2$	-	-	-	-	-	-	-
	4-Heptanol	8.48	889	589-55-9	$C_7H_{16}O$	7.50	-	-	12.06	-	-	-
	Ethanol	2.27	456	64-17-5	C_2H_6O	128.53	158.21	7.46	-	-	-	-
	2,5-Heptanediol	7.29	1214	70444-25-6	$C_7H_{16}O_2$	-	-	-	-	9.06	-	-
	1-Propanol	10.92	556	71-23-8	C_3H_8O	1.41	-	-	-	1.02	-	-
	1-Pentanol	5.53	767	71-41-0	$C_5H_{12}O$	-	1.53	7.46	59.00	2.18	0.64	7.59
	4-Methyl-1-hexanol	15.26	915	818-49-5	$C_7H_{16}O$	-	-	-	-	9.76	0.69	-
	2-Methyl-1-octanol	15.44	1127	818-81-5	$C_9H_{20}O$	-	-	62.79	2.37	-	-	-
Aldehydes	Furfural	7.23	835	98-01-1	$C_5H_4O_2$	5.27	4.14	0.63	2.05	6.16	4.30	86.07
	Heptanal	9.33	901	111-71-7	$C_7H_{14}O$	-	180.31	2.49	2.79	2.69	1.64	2.35
	2-Methylpentanal	3.44	765	123-15-9	$C_6H_{12}O$	23.15	-	3.27	23.22	2.13	3.41	3.03
	Octanal	12.70	1004	124-13-0	$C_8H_{16}O$	-	1.15	0.53	50.43	91.50	1.00	-
	Nonanal	16.13	1104	124-19-6	$C_9H_{18}O$	2.87	0.48	0.51	-	1.03	1.33	2.07
	(*E*)-2-Heptenal	11.15	956	18829-55-5	$C_7H_{12}O$	-	0.84	0.69	4.92	1.31	1.47	9.92

	(*E*)-2-Octenal	14.59	1061	2548-87-0	$C_8H_{14}O$	-	0.42	0.51	2.80	-	2.10	-
	3-Furaldehyde	38.77	836	498-60-2	$C_5H_4O_2$	-	-	-	-	-	-	-
	Butanedial	2.30	778	638-37-9	$C_4H_6O_2$	-	-	-	304.11	-	-	528.19
	Hexanal	6.29	801	66-25-1	$C_6H_{12}O$	27.94	0.58	0.78	4.27	6.36	3.70	55.70
	(*Z*)-3-Hexenal	7.88	799	6789-80-6	$C_6H_{10}O$	-	-	-	-	-	-	1.62
	(*E*)-4-Heptenal	13.58	955	929-22-6	$C_7H_{12}O$	-	-	-	2.46	-	-	-
	2-Methylbutanal	3.56	652	96-17-3	$C_5H_{10}O$	19.36	17.75	130.20	15.84	348.84	35.30	5.14
	2-Methylheptanoic acid	6.96	1167	1188-02-9	$C_8H_{16}O_2$	-	10.07	3.04	45.51	-	-	-
Acids	Oxalic acid	2.23	748	144-62-7	$C_2H_2O_4$	-	-	-	-	-	94.31	-
	2-Methyldecanoic acid	23.40	1455	24323-23-7	$C_{11}H_{22}O_2$	6.01	5.84	6.77	17.39	7.54	2.01	1.88
	2-Oxoglutaric acid	11.73	876	328-50-7	$C_5H_6O_5$	2.08	2.90	1.77	24.36	4.45	365.95	-
	Acetic acid	3.01	605	64-19-7	$C_2H_4O_2$	1.76	-	-	-	-	-	-
Esters	Methyl hexanoate	10.06	928	106-70-7	$C_7H_{14}O_2$	4.09	0.34	0.58	3.33	2.51	2.86	893.95
	Methyl formate	4.05	523	107-31-3	$C_2H_4O_2$	20.20	9.31	0.40	10.98	-	-	-
	Methyl octanoate	16.87	1128	111-11-5	$C_9H_{18}O_2$	2.40	1.01	-	12.05	1.14	4.58	16.12
	Methyl sorbate	13.18	1052	1515-80-6	$C_7H_{10}O_2$	2.00	10.19	0.98	179.35	2.90	0.95	4.78
	2-Isopropoxyethyl acetate	20.80	938	19234-20-9	$C_7H_{14}O_3$	-	-	-	-	-	-	1.70
	Butyl pentanoate	31.17	959	23361-78-6	$C_9H_{18}O_2$	4.81	-	-	4.15	-	1.29	-
	Methyl 4-methylpentanoate	9.02	855	2412-80-8	$C_7H_{14}O_2$	-	-	-	-	-	-	-
	Pentyl acrylate	11.83	1028	2998-23-4	$C_8H_{14}O_2$	-	-	-	-	-	-	3.30
	Vinyl pivalate	15.01	839	3377-92-2	$C_7H_{12}O_2$	-	0.55	-	-	-	-	-
	Isobutyl nitrate	4.72	763	543-29-3	$C_4H_9NO_3$	-	-	-	-	65.28	4.91	-
	Methyl isobutyrate	3.84	675	547-63-7	$C_5H_{10}O_2$	1.79	-	0.86	3.59	27.94	16.98	9.25
	Methyl propionate	5.71	635	554-12-1	$C_4H_8O_2$	22.76	3.42	20.48	50.47	1.83	9.77	88.02
	Methyl isovalerate	8.78	775	556-24-1	$C_6H_{12}O_2$	-	-	-	-	-	1.03	-
	Butyl propionate	2.79	899	590-01-2	$C_7H_{14}O_2$	-	-	-	-	-	-	39.34

	Methyl butyrate	4.49	717	623-42-7	$C_5H_{10}O_2$	3.58	0.72	11.31	7.25	2.16	2.63	46.14
	Methyl pentanoate	6.98	818	624-24-8	$C_6H_{12}O_2$	0.87	-	1.86	7.45	1.60	0.98	1.73
	Pentyl propionate	9.33	994	624-54-4	$C_8H_{16}O_2$	0.98	-	-	-	-	0.63	-
	Methyl nitrite	2.20	527	624-91-9	CH_3NO_2	-	-	-	-	-	-	256.18
	Methyl laurate	36.41	1491	627-90-7	$C_{13}H_{26}O_2$	-	-	-	89.71	2.35	-	-
	Methyl stearate	36.34	1990	628-97-7	$C_{18}H_{36}O_2$	37.92	15.24	1.65	-	-	27.82	-
	Vinyl formate	5.10	713	692-45-5	$C_3H_4O_2$	-	1.44	-	-	-	-	-
	Methyl 4-oxodecanoate	36.90	913	7011-82-7	$C_{11}H_{20}O_3$	-	-	-	3.96	-	-	-
	Ethyl 2-methylbutanoate	7.72	848	7452-79-1	$C_7H_{14}O_2$	-	-	0.88	-	-	-	-
	Methyl acetate	2.46	521	79-20-9	$C_3H_6O_2$	42.62	7.94	20.82	626.99	51.45	31.27	84.95
	Methyl 2-oxoacetate	8.64	787	922-68-9	$C_3H_4O_3$	-	0.39	-	-	9.72	-	-
	N-propyl acrylate	31.03	967	925-60-0	$C_6H_{10}O_2$	3.38	-	-	-	-	-	-
	Allyl formate	6.72	586	1838-59-1	$C_4H_6O_2$	-	-	-	-	-	-	-
ketones	2,4-Dimethyl-3-heptanone	11.68	752	18641-71-9	$C_9H_{18}O$	-	0.67	-	-	-	-	-
	4-Methyl-5-nonanone	6.09	1087	35900-26-6	$C_{10}H_{20}O$	3.81	-	-	-	-	-	-
	1-Octen-3-one	5.16	978	4312-99-6	$C_8H_{14}O$	8.76	3.52	9.64	-	51.45	12.51	-
	5-Methyl-4-octanone	11.67	988	6175-51-5	$C_9H_{18}O$	1.87	-	-	2.57	6.26	-	-
Alkenes	Myrcene	12.23	995	123-35-3	$C_{10}H_{16}$	2.73	-	-	-	-	-	-
	3-Carene	14.15	1008	13466-78-9	$C_{10}H_{16}$	-	1.59	0.37	-	-	0.74	-
	(1S)-(1)-*β*-Pinene	12.24	1007	18172-67-3	$C_{10}H_{16}$	3.06	2.09	0.44	2.37	1.21	4.08	8.16
	(*E*)-3-Decene	19.74	1025	19150-21-1	$C_{10}H_{20}$	-	0.82	-	-	-	-	1.88
	6-Methyl-1-heptene	8.37	754	5026-76-6	C_8H_{16}	5.10	0.71	2.46	8.02	1.73	-	-
	3-Methyl-1-pentene	11.16	545	760-20-3	C_6H_{12}	2.23	1.01	1.17	-	-	-	16.05
	α-Pinene	13.81	945	80-56-8	$C_{10}H_{16}$	-	-	-	-	-	0.88	-
	2-Methyloctane	15.17	866	3221-61-2	C_9H_{20}	-	-	0.51	2.58	5.01	9.34	-
	3,3-Dimethylhexane	28.83	761	563-16-6	C_8H_{18}	0.85	-	-	4.84	3.29	-	-

	Tridecane	30.23	1300	629-50-5	$C_{13}H_{28}$	8.76	3.25	0.49	-	-	-	-
	Pentadecane	27.47	1500	629-62-9	$C_{15}H_{32}$	2.52	-	0.78	-	-	-	-
	2-Methyldecane	27.67	1055	6975-98-0	$C_{11}H_{24}$	7.53	-	-	-	2.24	-	4.15
	2-Pentylfuran	12.26	990	3777-69-3	$C_9H_{14}O$	3.90	0.78	1.02	5.97	3.80	2.10	-
Other	Anethole	20.99	1280	104-46-1	$C_{10}H_{12}O$	28.14	2.99	-	-	-	-	-

RT: Retention time determined using an Elite-5MS capillary column (30 m × 0.25 mm × 0.25 μm).

“-”: not detected.

Supplementary Table S2. OAVs of volatile compounds in JR samples during ripening.

Compounds	CAS	Threshold (mg/kg)	Sample								Aroma descriptor
			JR0	JR5	JR10	JR15	JR20	JR25	JR30	JR35	
1-Hexanol	111-27-3	0.034	22.33	42.44	44.13	60.44	89.52	-	-	-	Green, grassy, fruity
3-Methyl-1-butanol	123-51-3	0.0061	964.42	1832.93	1939.48	-	-	-	-	-	Malty, alcoholic, fermented
1-Octen-3-ol	3391-86-4	0.0027	115.20	218.94	271.12	445.74	660.21	-	-	-	Mushroom, earthy
1-Hepten-3-ol	4938-52-7	0.01	64.34	122.29	100.95	128.26	126.65	1075.08	755.15	525.42	Green, earthy, mushroom
Ethanol	64-17-5	0.62	79.72	151.50	160.03	-	-	-	-	-	Alcoholic
1-Propanol	71-23-8	0.24	1.05	1.99	2.96	2.65	2.62	-	-	-	Alcoholic, pungent
1-Pentanol	71-41-0	0.36	4.64	8.82	8.53	9.75	8.08	31.82	109.14	75.94	Alcoholic, fermented
Furfural	1998/1/1	2.8	0.12	1.51	1.73	2.13	2.71	23.77	13.34	11.02	Caramel, almond
Heptanal	111-71-7	0.26	188.91	359.04	5.50	6.78	6.89	9.18	6.17	3.41	Fatty, green, citrus
2-Methylpentanal	123-15-9	0.4	2.20	4.18	25.69	31.67	29.75	29.93	32.19	22.40	Ethereal, fruity, green
Octanal	124-13-0	0.17	1.68	3.46	3.43	7.34	314.62	385.76	210.69	134.46	Citrus, fruity, fatty
Nonanal	124-19-6	0.0031	86.10	163.64	569.19	701.84	664.77	711.17	282.74	243.83	Citrus, floral, waxy
Hexanal	66-25-1	0.23	1.58	3.01	61.27	75.55	67.84	164.60	92.38	55.86	Green, grassy, fatty
(*Z*)-3-Hexenal	6789-80-6	0.0012	-	-	-	-	-	1019.02	571.90	3941.92	Fresh green, leafy, grassy
(*E*)-4-Heptenal	929-22-6	0.034	-	-	-	-	-	-	42.25	29.40	Fatty, fishy, green
2-Methylbutanal	96-17-3	1.1	63.61	42.46	42.06	66.88	117.94	14.40	8.08	1.34	Malty, cocoa, nutty
Acetic acid	64-19-7	0.013	24.28	46.15	68.58	-	-	-	-	-	Vinegar
Methyl hexanoate	106-70-7	0.07	3.51	6.68	47.49	58.55	55.55	7754.55	4352.07	2526.77	Fruity, pineapple
Methyl octanoate	111-11-5	0.2	-	5.18	12.13	17.85	17.62	98.18	55.10	36.20	Fruity, orange, wine
Methyl isobutyrate	547-63-7	0.007	33.05	62.81	258.17	2662.11	2628.64	3146.07	1664.93	963.75	Fruity, floral, apple, pineapple
Methyl propionate	554-12-1	0.35	18.35	34.87	38.68	47.69	40.32	257.28	144.39	89.65	Herbaceous, fruity
Methyl isovalerate	556-24-1	0.011	12.60	23.94	31.62	58.49	-	-	-	-	Earthy, faintly sweet
Butyl propionate	590-01-2	0.19	18.55	35.25	41.90	64.58	85.03	156.38	-	-	Apple-like, fruity

Methyl butyrate	623-42-7	0.03	90.98	172.91	168.38	196.87	167.52	1324.25	743.21	512.00	Green, apple, pineapple
Methyl pentanoate	624-24-8	0.011	90.82	71.94	71.27	92.26	103.01	164.45	162.09	76.19	Apple-like, fruity
Pentyl propionate	624-54-4	0.085	-	-	6.35	7.83	7.46	8.13	3.19	316.12	Fruity, apple
Methyl laurate	627-90-7	0.3	-	-	-	-	4.82	5.90	129.49	90.10	Fruity, fatty
Methyl stearate	628-97-7	2	4.34	8.25	8.68	12.89	-	-	-	-	Fatty, waxy
Methyl acetate	79-20-9	5.1	1.52	2.88	7.33	9.04	12.43	25.16	75.65	52.63	Fruity, ethereal
1-Octen-3-one	4312-99-6	0.012	294.77	560.23	738.55	1300.64	2112.48	2590.14	1211.38	1195.80	Mushroom, earthy, metallic
Myrcene	123-35-3	0.0012	406.86	773.27	1149.06	-	-	-	-	-	Herbal, balsamic, resinous
α-Pinene	80-56-8	0.1	-	-	-	3.92	3.87	4.43	1.86	5.47	Pine, resinous, woody
2-Pentylfuran	3777-69-3	0.27	4.71	8.94	9.11	11.94	12.81	16.61	9.37	-	Green, earthy
Anethole	104-46-1	0.057	64.32	122.24	184.17	-	-	-	-	-	Anise, licorice, sweet

Note: "-" indicates that the compound was not detected.
